# Impulsivity in Gambling Disorder and problem gambling: a meta-analysis

**DOI:** 10.1038/s41386-019-0393-9

**Published:** 2019-04-16

**Authors:** Konstantinos Ioannidis, Roxanne Hook, Katie Wickham, Jon E. Grant, Samuel R. Chamberlain

**Affiliations:** 1 Department of Psychiatry, University of Cambridge, Cambridge, CB2 0QQ UK; 2 Cambridge and Peterborough NHS Foundation Trust, Cambridge, UK; 3 Department of Psychiatry and Behavioral Neuroscience, University of Chicago, Chicago, IL USA

**Keywords:** Cognitive neuroscience, Human behaviour

## Abstract

Gambling Disorder is a prevalent psychiatric condition often linked to dysfunction of cognitive domains regulating impulsive behavior. Despite the centrality of impulsivity to neurobiological models of Gambling Disorder, a comprehensive meta-analysis of all impulsive cognitive domains has yet to be conducted. It is also not clear whether cognitive deficits in Gambling Disorder extend to those with problem (at-risk) gambling. A systematic review was undertaken of case–control studies examining the following cognitive domains in Gambling Disorder or in at-risk (problem) gambling: attentional inhibition, motor inhibition, discounting, decision-making, and reflection impulsivity. Case–control differences in cognition were identified using meta-analysis (random-effects modeling). Moderation analysis explored potential influences of age, gender, presence/absence of comorbidities in cases, geographical region, and study quality on cognitive performance. Gambling Disorder was associated with significant impairments in motor (g = 0.39–0.48) and attentional (g = 0.55) inhibition, discounting (g = 0.66), and decision-making (g = 0.63) tasks. For problem gambling, only decision-making had sufficient data for meta-analysis, yielding significant impairment versus controls (g = 0.66); however, study quality was relatively low. Insufficient data were available for meta-analysis of reflection impulsivity. There was evidence for significant publication bias only for the discounting domain, after an outlier study was excluded. Study quality overall was reasonable (mean score 71.9% of maximum), but most studies (~85%) did not screen for comorbid impulse control and related disorders. This meta-analysis indicates heightened impulsivity across a range of cognitive domains in Gambling Disorder. Decision-making impulsivity may extend to problem (at-risk) gambling, but further studies are needed to confirm such candidate cognitive vulnerability markers.

## Introduction

Gambling is a commonplace activity across the globe. While many people engage in gambling recreationally without marked negative personal consequences, some individuals develop maladaptive symptoms of disordered gambling, which may ultimately manifest as full Gambling Disorder (also known as Pathological Gambling), characterized by functional impairment. Gambling Disorder is associated with untoward longer-term outcomes, including reduced quality of life, and elevated risk of divorce, financial issues (bankruptcy/insolvency), and incarceration [1]. Gambling Disorder was previously included in the Diagnostic and Statistical Manual Version IV (DSM-IV) as an impulse control disorder not elsewhere classified, but was moved in DSM-5 to the category of Substance Related and Addictive Disorders [2]. It shares parallels with impulse disorders and substance disorders from several vantage points, including in terms of phenomenology, comorbid overlap, and neurobiological models [3–5].

The concept of impulsivity is central to understanding Gambling Disorder and related addictions [6], and was highlighted as an important overarching construct in a recent Delphi analysis [4]. Impulsivity refers to behaviors or acts that are unduly hasty, risky, and inappropriate, leading to negative outcomes [7]. Recent models of impulsivity highlight its complex, multifactorial nature, and the need to consider not only its behavioral manifestations but also underlying brain-based and psychological mechanisms [8, 9]. As noted, Gambling Disorder was previously listed alongside impulse control disorders; furthermore, elevated impulsivity at the level of personality traits and occurrence of impulse control disorders is found in Gambling Disorder and family members, suggesting that some elements, at least of impulsivity, may be familial, and may be regarded as vulnerability markers [10, 11]. From a cognitive perspective, impulsivity can be fractionated into different domains, including impulsive choice (preference for smaller more immediate rewards rather than larger delayed rewards), impulsive motor responses (failure to suppress inappropriate motor responses), impulsive decision-making (risky/suboptimal choices under situations of ambiguity), reflection impulsivity (tendency to make premature responses to solutions under conditions of high response uncertainty), and impulsive cognitive bias (failure to suppress inappropriate attentional bias) [12–15]. Although these cognitive domains appear to be in many cases partly dissociable from each other, both behaviorally [12], and in terms of neurochemical substrates across species [7, 16], they tend to co-occur at the latent phenotype level of conceptualization [17]. Meta-analytic studies have identified impulsivity in Gambling Disorder in some of these cognitive domains viewed individually [18, 19]. However, analysis across the full range of domains is lacking, and so it is not well-established whether disordered gambling is associated with particular circumscribed deficits or more generalized inhibitory dyscontrol. It is also not yet established from meta-analysis whether impulsive cognitive dysfunction extends to people with some degree of disordered gambling falling short of the full diagnosis (termed “at-risk” or “problem” gambling). Furthermore, effects of moderators such as study quality on impulsive cognition have not been rigorously examined.

The aim of this paper was to conduct a comprehensive meta-analysis of the range of cognitive domains relevant to impulsivity in Gambling Disorder, including examination of key moderators. Domains of interest were: attentional inhibition, motor inhibition, discounting, decision-making, and reflection impulsivity. Furthermore, we evaluated datasets not only for Gambling Disorder but also for problem gambling (defined as datasets for which the case group included disordered gamblers not meeting the diagnostic threshold). We hypothesized that Gambling Disorder would be associated with elevated impulsiveness across all domains; but that decision-making impairment would also extend to problem gambling, consistent with impaired decision-making being a candidate “early” vulnerability marker as suggested by some prior case–control research [20].

## Materials and methods

The study followed the Meta-analysis Of Observational Studies in Epidemiology (MOOSE) guidelines [21], and the Protocol was registered electronically and published online on the PROSPERO International prospective register of systematic reviews, prior to data analysis (https://www.crd.york.ac.uk/prospero/display_record.php?RecordID=105900).

### Search strategy

The initial literature search string was determined through consensus between the study authors, based on expert knowledge of Gambling Disorder and neuropsychological assessment. Two members of the study team conducted a PubMed search for papers published in English, using the following string: [“cognitive” OR “cognition” OR “neuropsychological tests” OR “memory” OR “executive” OR “attention” OR “decision-making” OR “gambling task” OR “Iowa Gambling” OR “Bechara Gambling” OR “Cambridge Gamble” OR “Cambridge Gambling” OR “Balloon analogue” OR “N-Back” OR “pointing task” OR “tapping” OR “tower of London” OR “stockings of Cambridge” OR “Wisconsin Card” OR “ID/ED” OR “Set-shifting” OR “Intra-dimensional” OR “intradimensional” OR “extra-dimensional” OR “extra dimensional” OR “inhibition” OR “stroop” OR “stop-signal” OR “go no go” OR “go/no-go” OR “gng”] AND [“Gambling Disorder” OR “pathological gambling” OR “problem gambling” OR “compulsive gambling” OR “gambling addiction” OR “gambling addictions” OR “problematic gambling” OR “pathological gamblers” OR “problem gamblers” OR “gamblers anonymous” OR “gambling addicts”]. We included search terms related to domains other than impulsivity in order to maximize detection of potentially relevant papers (since studies often examine multiple domains). From the identified papers, those obviously out-of-scope were identified and discarded by a member of the research team, based on reading of the abstract. Review papers were scanned for additional potentially relevant data papers by reading reference lists. Papers identified as potentially out-of-scope were discussed in a meeting comprising four members of the study team, to arrive at a consensus decision. Identified data papers were then obtained and read by a member of the research team, those obviously out-of-scope were excluded. Data papers potentially out-of-scope were discussed in a meeting comprising at least four members of the study team, again to arrive at a consensus decision, based on reading the full paper.

### Inclusion criteria

We included all studies that (a) were published in scholarly peer-review journals between 1987 and December 2018; (b) were written in English or provided an English translation; (c) examined cognitive measures in controls versus participants with at least some degree of disordered gambling i.e., meeting at least some Diagnostic and Statistical Manual Version III-R (DSM-III-R) or subsequent diagnostic criteria (i.e., DSM-III-R or DSM-IV or DSM-5); and (d) there was available information to calculate an effect size. In instances where insufficient data were reported in a given data paper (e.g., data presented only in graphical format; or standard deviation missing), the authors of these data papers were contacted via e-mail to request this information. The full list of included papers can be found in the supplement (Table S1).

### Exclusion criteria

**Fig. 1:**
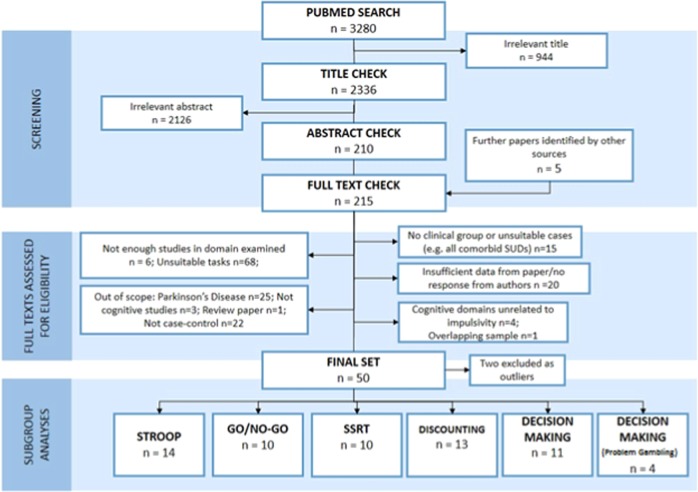
PRISMA flow diagram—Meta-analysis of Impulsivity in Gambling Disorder

We excluded studies that (a) did not report cognitive measures; (b) used non-standard cognitive tasks unsuited to meta-analysis; (c) did not have a healthy comparison group; (d) examined a specific domain where there were less than four studies examining the given cognitive domain for the group of interest; (e) used measures not reported in the full text, and for which the authors did not reply in a timely manner (at least 4 weeks given) to provide the necessary data; (f) were published only in the gray literature (including conference papers, non-peer reviewed publications, doctoral theses etc.); and (g) focused on neurologic disorders such as Parkinson’s Disease (as the pathology of gambling in such cases is non-typical, e.g., due to dopaminergic medication). Our rationale for excluding gray literature was to avoid inclusion of studies that had not gone through appropriately rigorous peer review. The process of exclusion is outlined in Fig. 1 (PRISMA Flowchart). The full list of excluded papers at the final stage are presented in the supplement (Table S2).

### Data extraction

Data from the resulting final list of papers were extracted. Information from the included studies was recorded in an electronic spreadsheet, including conventional data needed for meta-analysis (task performance mean, SD, N per group) along with: (a) broad geographical region, in which the data collection occurred (“Asia”, “Europe”, etc.); (b) key demographics of the participants (age as categorized by mean age reported in the sample: children 0–12, youth 12–24, adults 24–55, older people ≥ 55); gender distribution in the sample (“male only”, “female only”, or “mixed”); (c) category of gambling problems (Gambling Disorder, i.e., meeting conventional thresholds consistent with a diagnosis [at least four diagnostic criteria or equivalent]; or problem gambling, i.e., meeting at least some diagnostic criteria); (d) presence or absence of psychiatric comorbidities in the cases (if reported); (e) quality scores (range 0–8 points). For each cognitive task, the task outcome measure of interest was selected based on previous literature, established norms in the field, and consensus among the whole study team. Within each cognitive domain, we included one measure of interest from each given study, thereby avoiding this issue of including non-independent variables within a given analysis. The quality scores were determined as follows: determination of gambling symptom extent / threshold using a validated instrument (e.g., SOGS or other structured instrument), 1 point; report and/or exclusion of psychiatric comorbidities (e.g., depression, psychosis) using a validated instrument (e.g., Structured Clinical Interview, SCID; e.g., Mini-International Neuropsychiatric Inventory, MINI), 1 point; report and/or exclusion of substance use disorder(s) (e.g., Structured Clinical Interview, SCID), 1 point; actual exclusion of comorbid SUDs using reasonable steps e.g., urine drug screen and/or psychiatric assessment, 1 point; report and/or exclusion of impulse control disorders using appropriate valid instrument (e.g., Minnesota Impulse Disorders Inventory, MIDI), 1 point (0.5 points were given for partial screening such as documenting history of ADHD or antisocial personality disorder); measurement of IQ and/or educational level, 1 point; report of suitable cognitive task outcome measures (as opposed to secondary measures), 1 point; numerical report contained in paper necessary for meta-analysis (i.e., mean, variance, N), 1 point (0 points if authors had to request these data). The full quality results for all papers are presented in the supplement (Table S3).

### Data analysis

Meta-analysis was conducted where at least four datasets were available for a given type of cognitive task relevant to impulsivity, for a given group of interest (Gambling Disorder or problem gambling), with the measure of interest being hedge’s g. For convenience, all meta-analysis plots were shown such that positive values on the X-axes indicated higher impulsivity (worse performance) in the gambling group compared with the control group. We first conducted an exploratory analysis of influence to identify outliers. During this process, we excluded two studies that were identified as highly influential and were of relatively low study quality (Cook’s d influence > 2 SD above domain mean and quality score < 15% of sample; [22] [Stroop] and [23] [Discounting]). Full results including those outliers are presented in the supplement (Fig. S2a-c). There was significant heterogeneity in all domains apart from decision-making (both in pathological and problem gamblers) [see Supplement Table S4]). Despite the lack of heterogeneity in the decision-making domains (both pathological and problem gambling Q-test *p*-value > 0.05), a random-effects model (REM) was used in all cases to provide a more generalizable model estimate. This decision was based on the fact that often the Q-test is underpowered in most meta-analyses, but furthermore on the assumption that those studies included in the meta-analysis sampled populations that differed in ways (e.g., study quality, presence of comorbidities) that could potentially impact on the observed effects. Full measures of heterogeneity for all domains included are presented in the supplement [Table S4]. Data were analyzed using statistical software R version 3.4.2. Meta-analysis was performed using packages of “robumeta” and “metafor” [24]. The R code used for this analysis is shared in the supplement to support reproducible research. Moderator analysis (all models were meta-regression) was conducted for age, gender, presence of comorbidities, geographical region, and quality scores. In the interest of space, references for all data papers were provided in the supplement rather than paper.

## Results

In total, 52 independent studies were included in the meta-analysis. The average quality score was 5.75/8 (71.9%).

### Stroop attentional inhibition

**Fig. 2:**
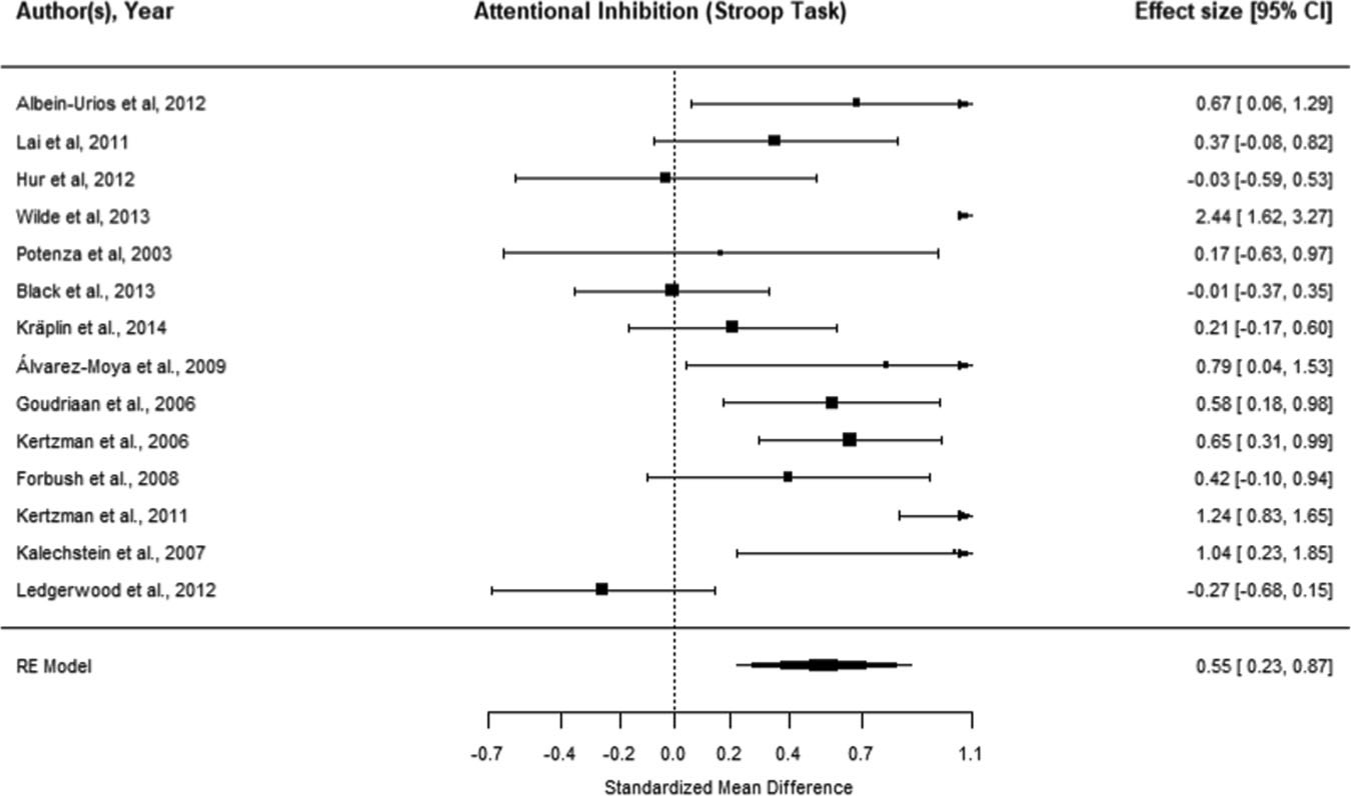
Meta-analysis of color-word Stroop attentional impulsivity in Gambling Disorder compared with controls. “Worse in gambling” indicates lack of attentional inhibition (e.g., higher color-word interference cost) in cases compared with controls

Sixteen datasets were identified: 15 in Gambling Disorder and one in problem gambling (not considered further). Regard et al. [22] were excluded as an outlier (Cook’s d influence > 2 SD and Quality score < 15% of sample). Meta-analysis of the 14 Gambling Disorder datasets (*N* = 464 cases, *N* = 575 controls) indicated that Gambling Disorder was associated with significant Stroop attentional impulsivity (g = 0.55 [CI: 0.23–0.87], *p* = 0.001; Fig. 2). Moderation analysis did not indicate any significant effect of gender, geographical location, presence of comorbidities, or study quality (see Supplement Tables S5, S6). Age could not be examined due to lack of comparison groups. Funnel plot test for asymmetry did not indicate evidence of publication bias. By excluding [22] as an outlier, we present a more conservative effect size (Hedge’s g 0.55 vs. 0.76), we addressed moderation by study quality (*p* = 0.24 vs. 0.0019) and addressed publication bias (*p* = 0.06 vs. *p* < 0.001).

### Go/No-Go

**Fig. 3:**
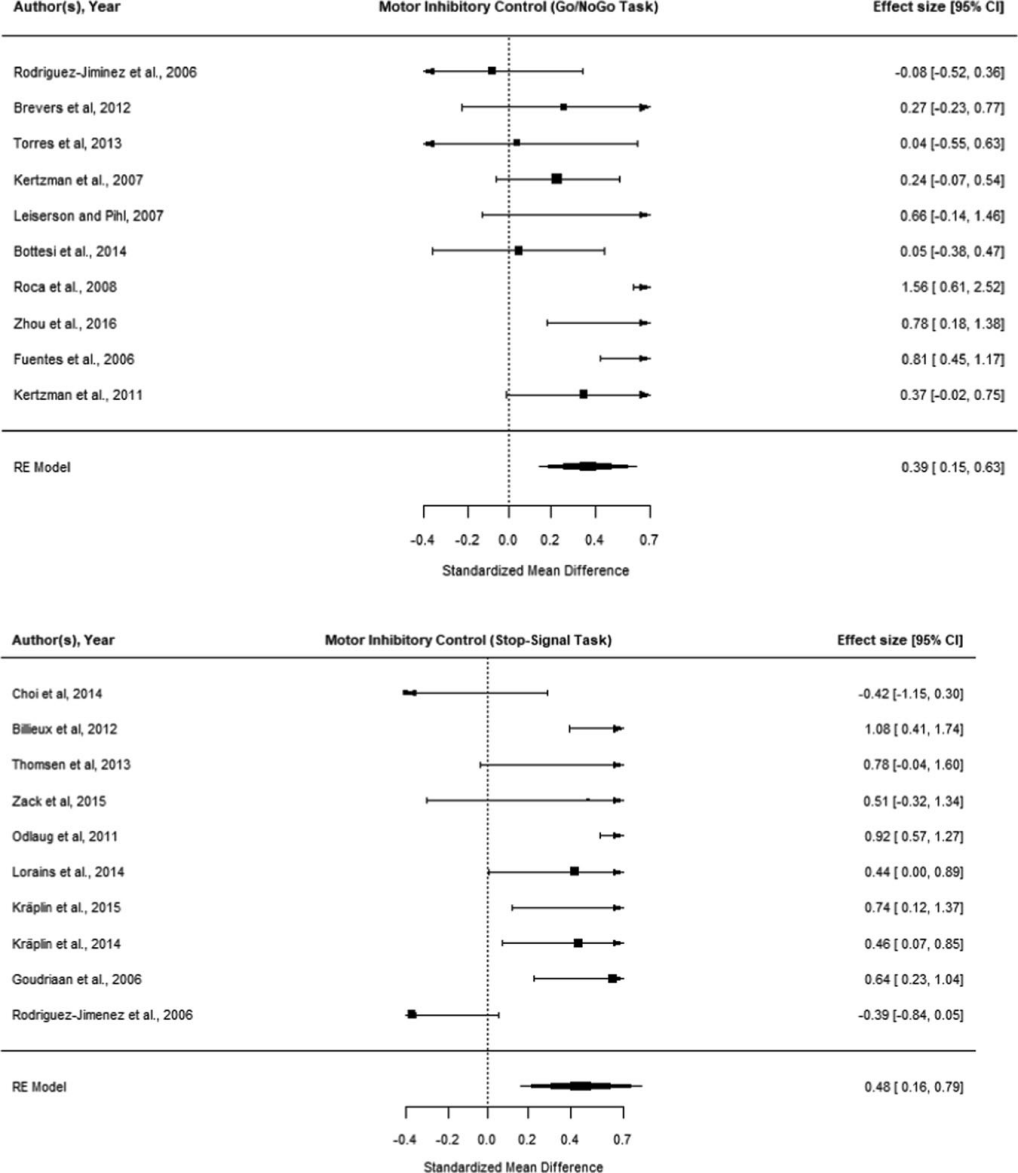
Meta-analysis of motor inhibition in Gambling Disorder, compared with controls, using Go/No-Go (top) and Stop-Signal (bottom) tasks. “Worse in gambling” indicates more commission errors (top) or longer Stop-signal reaction times (bottom)

Total of 12 datasets were identified: 10 for Gambling Disorder and two for problem gambling (not considered further). Meta-analysis (*N* = 358 cases, *N* = 417 controls) identified a significant Go/No-Go inhibitory deficit in Gambling Disorder (g = 0.39 [CI: 0.15–0.63], *p* < 0.001; Fig. 3, top). Moderation analysis did not indicate any significant effects of age, gender, study quality, or presence of comorbidities. However, effect sizes were moderated by geographical area (Asia < Europe). Inspection and test for plot asymmetry of the funnel plot did not identify publication bias (Supplementary Fig. S1).

### Stop-Signal task

Fourteen datasets were identified: 10 in Gambling Disorder and four in problem gambling. Of the problem gambling studies, two had overlapping data, leaving three unique datasets, hence meta-analysis was not undertaken. For the 10 Gambling Disorder datasets (*N* = 298 cases, *N* = 428 controls), there was significant response inhibition impairment in Gambling Disorder (g = 0.48, [CI: 0.16–0.79] *p* = 0.003; Fig. 3, bottom). Moderation analysis indicated a significant effect of gender (*p* = 0.003); and of study quality (*p* = 0.029). Mixed as opposed to all-male gender studies were associated with worse cognitive performance in cases, and higher study quality was associated with more pronounced cognitive deficits in cases. There was no significant effect of geographical location or comorbidities. Age could not be examined due to lack of comparison groups (see Supplement Table S5). Inspection of the funnel plot and test for asymmetry did not indicate evidence of publication bias (Supplementary Fig. S1).

### Discounting

**Fig. 4:**
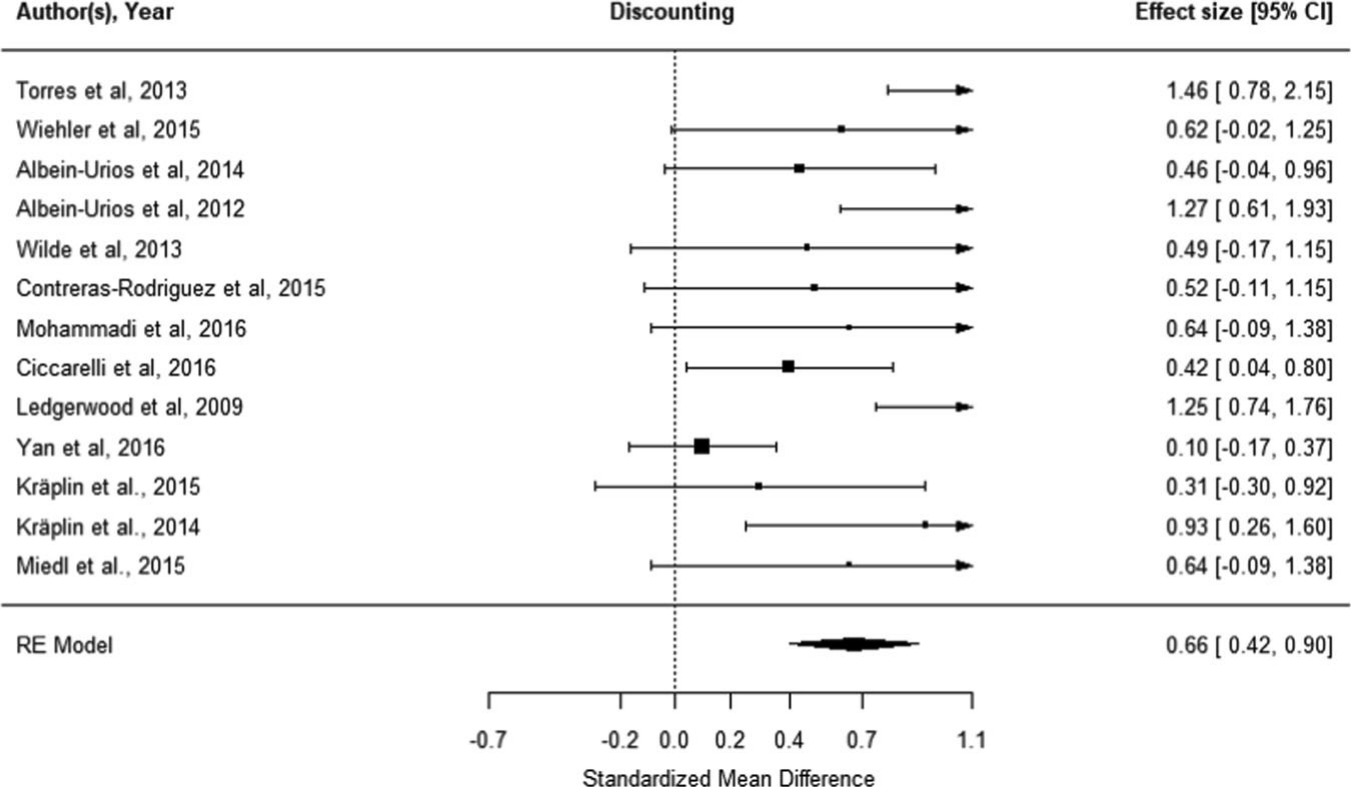
Meta-analysis of discounting task performance in Gambling Disorder compared with controls. “Worse in gambling” indicates steeper discounting/relative preference for smaller more immediate rewards in the cases versus controls

In total, 17 datasets were identified: 14 for Gambling Disorder and three for problem gambling (not considered further). Wiehler et al. [23] were excluded as an outlier (Cook’s d influence > 2 SD and Quality score < 15%). Across 13 datasets (*N* = 326 cases, *N* = 1323 controls), Gambling Disorder was associated with elevated discounting impulsivity (g = 0.66 [CI: 0.42–0.90], *p* < 0.001; Fig. 4). Moderation analyses identified that adult studies reported higher estimates than the youth study [25]; effect sizes were moderated by geographical area (Asia < Europe < USA). We did not identify any moderation from gender, study quality, or presence of comorbidities (see Supplement Tables S5, S6). The funnel plot test for plot asymmetry identified evidence of publication bias (z = 2.26, *p* = 0.02; Supplementary Fig. S1). Results with the inclusion of the outlier study are presented in the supplement (Fig. S2b). By excluding this outlier, we report a more conservative effect size (Hedge’s g 0.66 vs. 0.86) and identified underlying moderation effects of age (*p* = 0.03 vs. *p* = 0.26) and geographic area (*p* = 0.007 vs. *p* = 0.48), as well as publication bias (*p* = 0.02 vs. 0.06).

### Decision-making tasks

**Fig. 5:**
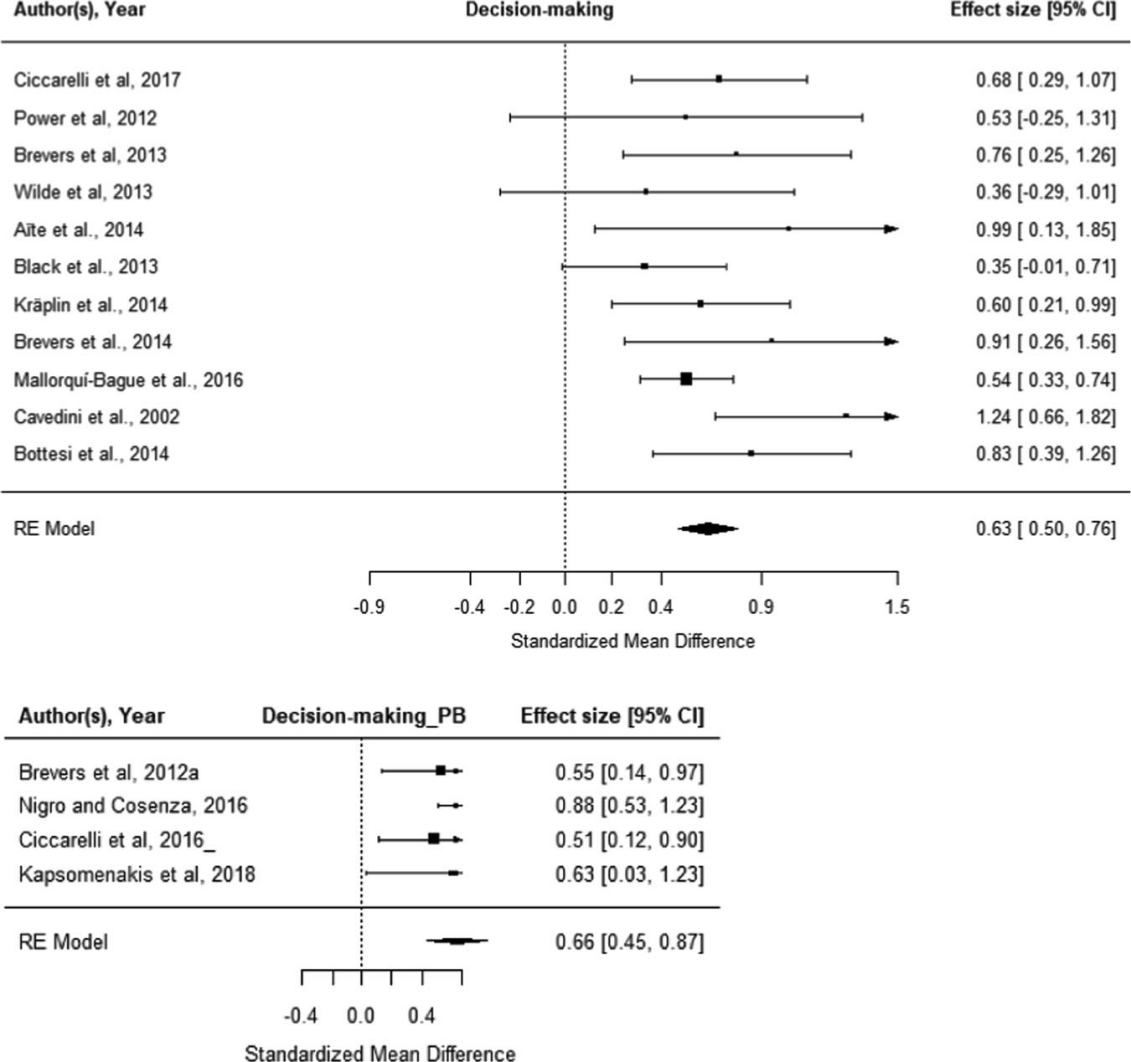
Meta-analysis of IGT decision-making in Gambling Disorder, compared with controls (top); and in problem gambling, compared with controls (bottom). “Worse in gambling” indicates impaired decision-making (e.g., lower preference for advantageous card decks) in cases compared with controls

There were insufficient data studies for meta-analysis of CGT performance, or BART performance. Fifteen datasets were identified for the IGT: 11 in Gambling Disorder and four in problem gambling. Meta-analysis indicated that Gambling Disorder (*N* = 493 cases, *N* = 560 controls) was also associated with impaired IGT decision-making (g = 0.63, [CI: 0.50–0.76]; *p* < 0.001; Fig. 5, top). Meta-regression did not indicate any significant effects of gender, geographical location, study quality, or presence of comorbidities. Age could not be examined due to lack of comparison groups. Inspection of the funnel plots and plot test for asymmetry did not indicate publication bias (Supplementary Fig. S1).

Meta-analysis indicated that problem gambling (*N* = 210 cases, *N* = 177 controls) was associated with impaired IGT decision-making (g = 0.66, [CI: 0.45–0.87]; *p* < 0.001; Fig. 5, bottom). Moderation analysis did not indicate a significant effect of age, geographical location gender, or study quality. Moderation of the presence of comorbidities could not be examined due to lack of comparison groups. Notably, influential studies were of low quality and the mean study quality was low (mean study quality score 3.75/8, at 15th centile of sample).

## Discussion

This study undertook a comprehensive meta-analysis of cognitive findings germane to impulsivity in Gambling Disorder, and in problem gambling (individuals fulfilling some but not necessarily all diagnostic criteria for Gambling Disorder), versus controls. The main finding was that Gambling Disorder was associated, in meta-analysis, with elevated impulsivity on motor inhibition, attentional inhibition, discounting, and decision-making tasks. These results were generally of medium effect size, except for Go/No-Go task motor inhibition, which was of small effect size. This analysis provides the first meta-analytic support for the existence of impulsivity in Gambling Disorder across cognitive domains, in keeping with neurobiological models implicating impulsivity and dysregulation of related frontostriatal brain pathways in the pathophysiology of disordered gambling [5, 26–28]. Thus, in fully established Gambling Disorder, impulsivity is evident across the full swathe of relevant cognitive tasks. These data also demonstrate elevated decision-making impulsivity (medium effect size) even in those with Problem Gambling, highlighting also the relative lack of studies on impulsivity in this context, and the need for further research. This is important because psychological models emphasize a likely role for impulsivity, as measured by behavioral measures, in the development—i.e., in the chain of pathogenesis—of Gambling Disorder [29, 30]. The concept of impulsivity also has broader relevance to other candidate behaviorally addictive disorders that are not currently listed in the DSM [4, 26, 31].

The finding of significant impairments across different impulsivity domains in Gambling Disorder has several potential interpretations. One interpretation is that distinct cognitive domains are independently impaired in Gambling Disorder, with each impairment having a different biological substrate in terms of fronto-striatal circuitry. Another interpretation, which we feel more likely per the law of parsimony, is that these findings reflect the existence of impulsivity at the latent phenotypic level for Gambling Disorder. Put differently, we hypothesize that there is a generalized tendency toward hasty, inappropriate, and premature actions, which predisposes toward Gambling Disorder and different manifestations of impulsivity across cognitive tasks. This may account for the common clinical observation that impulsive problems tend to co-occur within the same individual; and for multiple measures of impulsivity (behavior, cognition, and personality) exhibiting correlations at the population level [17]. In prior research, we found that 33 impulsive and compulsive problem behaviors were optimally explained statistically within a bifactor model of latent phenotypes: i.e., by a general factor (termed “disinhibition”) contributing to the full range of problem behaviors; and then two separable impulsive and compulsive factors more directly linked to the expression of particular problems [32]. Indeed such latent phenotypes have been associated with changes in functional connectivity between the basal ganglia and cortices, including in people with Gambling Disorder [33]. These prior latent phenotype studies did not examine cognition. The current meta-analysis suggests that it would be valuable to extend a bifactor model to impulsivity-related cognitive domains in Gambling Disorder, to test our above hypothesis. Identification and affirmation of such latent phenotypes may be valuable both in order to better understand common neurobiological mechanisms across addictive disorders, and also with a view to identifying early treatment targets.

The overall quality scores of the included studies was 71.9%, and we found no evidence, in moderation analyses, that worse study quality was associated with more pronounced cognitive deficits in any domain. The most common methodological issue (evident in 85% of the data studies) was failure to screen for impulse control and related disorders (including ADHD) using adequate instruments. Such conditions are often associated with impulsive cognitive problems in themselves and so may thus contribute to the neuropsychological profiles observed herein.

Turning to the other moderation variables, presence or absence of comorbidities in cases did not significantly affect the cognitive findings. We did not identify significant moderating effects of study age category, except for evidence that discounting deficits were more pronounced for adult studies than for the available youth study. It may be that case–control differences for this domain are harder to detect in younger samples due to increased noise arising from heterogeneous stages of brain development, as compared with the mature adult brain. The only significant moderating effect of gender was that studies including mixed (male and female) participants had larger Stop-signal inhibition deficits than studies including only males. We did not find gender-related differences in Stop-signal inhibition in a large pooled analysis previously, hence this result may be spurious [34]. Geographical location moderated the cognitive findings in several ways: Go/No-Go motor inhibition task deficits were larger in European studies compared with Asian studies; and discounting task deficits were larger for USA studies than for European studies, which in turn were larger than for Asian studies. The underlying reasons for these cross-cultural effects are unclear. Overall, this may suggest less marked case–control differences in cognition for data studies arising from Asia. Notable cross-cultural differences in rates of comorbidity, and the nature of gambling activities engaged in, have been reported in other settings for Gambling Disorder [35]. Direct head-to-head comparisons of impulsivity data from different geographical sites would be warranted in light of these moderation effects, in future work.

Though this is the first comprehensive meta-analysis in Gambling Disorder covering the broad range of cognitive domains related to impulsivity, several limitations should be noted. We focused on recognized well-validated cognitive measures suitable for meta-analysis, for which there were at least four datasets available for meta-analysis. As such, this is not an exhaustive analysis of all conceivable tasks or domains. We were not able to examine all types of decision-making tasks, due to lack of data, and our a priori specified choice of methodology for classifying such tasks. However, it is important to note that data studies have reported decision-making deficits across other tasks in Gambling Disorder; viz with the Cambridge Gambling Task, Balloon Analogue Risk Task, and the Game of Dice Task [36–38]. Selection of task measures of interest was based on expert consensus within the study team. Each meta-analysis included one measure of interest from a given domain per study, in order to avoid interdependence across variables within a given meta-analysis. Nonetheless, the separate meta-analyses within this paper cannot be regarded as being independent from each other, since in some cases they included data from several cognitive domains from a given data study. This approach is widely accepted for meta-analysis [39, 40], but does highlight the need for future data papers to examine the latent structure of impulsivity in Gambling Disorder (i.e., the possibility of latent phenotypes). We did not examine task measures related to emotional processing or bias for gambling stimuli; these tasks tend to be tailored for particular studies or participant populations and therefore are problematic for valid meta-analysis. For quality scores, we included whether comorbidities were appropriately identified; rather than whether individual data papers conducted statistical analyses to control for such potential confounds. This was for pragmatic purposes, since whether papers identified comorbidities is relatively easy and objective to assess; whereas evaluating whether control for identified comorbidities was appropriate is difficult to assess. For example, one could covary for ADHD symptoms in terms of a cognitive measure, but this relies on various statistical assumptions that may or may not be met, which we could not assess (including whether such analyses were sufficiently powered). For convenience, Gambling Disorder was defined using accepted cutoffs, but not all data papers used structured clinical interviews to make this diagnosis. The problem gambling datasets included, in some cases, potentially mixed samples (i.e., where some individuals of the group may have met the criteria for Gambling Disorder). Nonetheless the finding of decision-making impairment in this group in meta-analysis is consistent with a previous study using a different task, which found deficits in at-risk gamblers none of whom met the diagnostic threshold [20]. The moderation analyses were not always conducted due to lack of datasets in given categories; and it should also be considered that the number of datasets in the explored moderator categories was small in many cases, potentially limiting power to detect effects of moderators. In particular, more research is needed to examine how the cognitive profile of Gambling Disorder overlaps with, or differentiates from, other disorders associated with impulsivity, including personality disorders [41], but also impulse control and developmental disorders.

In summary, this meta-analysis found evidence for deficits in Gambling Disorder across all evaluable domains of impulsivity that were considered. Thus, in considering cognitive findings in this disorder, it is necessary to consider both impulsive and compulsive features [42]. In fully established Gambling Disorder, there is generalized impulsivity across the full range of domains. By contrast, decision-making impairment was also found in problem gambling, but there were insufficient data studies to address other cognitive domains. Thus, in keeping with neurobiological models and consensus views on Gambling Disorder, impulsivity is core to understanding Gambling Disorder. The extent to which these findings relate to vulnerability versus chronicity merits further study [4], as does the issue of the existence of latent phenotypes. The finding of decision-making deficits in at-risk gamblers here using the Iowa Gambling Task, and in a prior data study using a different task [20], indicates this is a particularly promising domain for identifying vulnerability markers in this setting.

## Funding and disclosure

Dr. Chamberlain’s involvement in this project was funded by a Wellcome Trust Fellowship (110049/Z/15/Z). Dr. Ioannidis’ research is supported by a CLAHRC Fellowship East of England (Collaboration in Leadership in Applied Health research and Care). Dr. Chamberlain consults for Cambridge Cognition, Promentis, and Shire; he receives a stipend for his role as Associate Editor at Neuroscience and Biobehavioral Reviews. Dr. Grant has received research grants from NIDA, National Center for Responsible Gaming, TLC Foundation for BFRBs, American Foundation for Suicide Prevention, Brainsway, and Psyadon and Takeda Pharmaceuticals. He receives yearly compensation from Springer Publishing for acting as Editor-in-Chief of the Journal of Gambling Studies and has received royalties from Oxford University Press, American Psychiatric Publishing, Inc., Norton Press, and McGraw Hill. The other authors report no disclosures or potential conflicts of interest.

## Supplementary information

Supplementary Material
