# Supplementary material for: Impulsivity in Gambling Disorder and problem gambling: a meta-analysis
Source: Neuropsychopharmacology. 2019 Apr 16;44(8):1354–61. doi: 10.1038/s41386-019-0393-9 (PMC6588525; doi:10.1038/s41386-019-0393-9)
Supplement: Supplementary file 1 — Supplementary Material [file 41386_2019_393_MOESM1_ESM.docx]

Supplementary Online File – Meta-analysis in Gambling

## TABLE S1 - Full list of data studies included in the meta-analysis

| PAPER | PMID | Age (Children, Youth, Adults, or Older People) | Gender (Mixed, male only, or female only) | Geographical Location (Europe, Asia, USA, other) | GD group type (GD or PG) | Instrument(s) used to determine GD status | Co-morbidities in patient group? (yes, no, or unclear) | | Cognitive Task |
| --- | --- | --- | --- | --- | --- | --- | --- | --- | --- |
| Aïte et al., 2014 | 24968006 | Adults | Mixed | Europe | GD | DSM-IV, SOGS (5 or over) | No | IGT | |
| Albein-Urios et al, 2012 | 22475814 | Adults | Unclear | Europe | GD | SCID-PG (Meeting DSM IV Criteria) | No | Stroop | |
| Albein-Urios et al, 2012 | 22475814 | Adults | Unclear | Europe | GD | SCID-PG (Meeting DSM IV Criteria) | No | Discounting | |
| Albein-Urios et al, 2014 | 25047890 | Adults | Unclear | Europe | GD | SCID-PG | No | Discounting | |
| Álvarez-Moya et al., 2009 | 19203440 | Adults | Females | Europe | GD | SOGS | No | Stroop | |
| Billieux et al, 2012 | 22421073 | Adults | Mixed | Europe | GD | SOGS (Over 5 for PGs) | Yes | SST | |
| Black et al., 2013 | NA | Adults | Mixed | USA | GD | NODS, SOGS (5 or over) | No | Stroop | |
| Black et al., 2013 | NA | Adults | Mixed | USA | GD | NODS, SOGS (5 or over) | No | IGT | |
| Bottesi et al., 2014 | 24863627 | Adults | Mixed | Europe | GD | SOGS | Yes | GNG | |
| Bottesi et al., 2014 | 24863627 | Adults | Mixed | Europe | GD | SOGS | Yes | IGT | |
| Brevers et al, 2012 | 23209796 | Adults | Mixed | Europe | GD | SOGS (5 or over) | No | GNG | |
| Brevers et al, 2012a | 22521232 | Adults | Mixed | Europe | PG | SOGS (5 and over for PG) | No | IGT | |
| Brevers et al, 2013 | 22273773 | Adults | Mixed | Europe | GD | SOGS (French version) | No | IGT | |
| Brevers et al., 2014 | 24980287 | Adults | Mixed | Europe | GD | SOGS | Unclear | IGT | |
| Cavedini et al, 2002 | 11958785 | Adults | Mixed | Europe | GD | SOGS (5 and over for PG) | No | IGT | |
| Choi et al, 2014 | 25592310 | Adults | Males | Asia | GD | PGSI (DSM-IV Criteria) | Unclear | SST | |
| Ciccarelli et al, 2016 | 26894884 | Youth | Males | Europe | PG | SOGS-RA (4 and over for problem gambling) | Unclear | IGT | |
| Ciccarelli et al, 2016 | 26846482 | Adults | Males | Europe | GD | DSM-5 criteria (endorsing 5 or 9 of the criteria) | No | Discounting | |
| Ciccarelli et al, 2017 | 27592413 | Adults | Males | Europe | GD | SOGS (5 and over for PG) | No | IGT | |
| Contreras-Rodriguez et al, 2015 | 26212416 | Adults | Mixed | Europe | GD | Unclear | No | Discounting | |
| Forbush et al., 2008 | 18408650 | Adults | Mixed | USA | GD | SOGS | Unclear | Stroop | |
| Fuentes et al., 2006 | 17064453 | Adults | Mixed | Other | GD | DSMI-IV, SOGS (5 and over) | No | GNG | |
| Goudriaan et al., 2006 | 16548933 | Adults | Mixed | Europe | GD | SOGS, SIDI | Yes | SST | |
| Goudriaan et al., 2006 | 16548933 | Adults | Mixed | Europe | GD | SOGS, SIDI | Yes | Stroop | |
| Hur et al, 2012 | 22794118 | Adults | Mixed | Asia | GD | SOGS (5 and over for PG) | No | Stroop | |
| Kalechstein et al., 2007 | 17827415 | Adults | Mixed | USA | GD | SCID-IV | No | Stroop | |
| Kapsomenakis et al, 2018 | 29455442 | Adults | Males | Europe | PG | SOGS (4 and over for probably PG) | No | IGT | |
| Kertzman et al., 2006 | 16626810 | Adults | Mixed | Asia | GD | SOGS (5 or over) | No | Stroop | |
| Kertzman et al., 2007 | 18789539 | Adults | Mixed | Asia | GD | SOGS (5 or over) | No | GNG | |
| Kertzman et al., 2011 | 21429591 | Adults | Mixed | Other | GD | SOGS (5 or over) | No | Stroop | |
| Kertzman et al., 2011 | 21429591 | Adults | Mixed | Other | GD | SOGS (5 or over) | No | GNG | |
| Kräplin et al., 2014 | 24930455 | Adults | Mixed | Europe | GD | DSM-IV | No | SST | |
| Kräplin et al., 2014 | 24930455 | Adults | Mixed | Europe | GD | DSM-IV | No | Stroop | |
| Kräplin et al., 2014 | 24930455 | Adults | Mixed | Europe | GD | DSM-IV | No | IGT | |
| Kräplin et al., 2014 | 24434041 | Adults | Males | Europe | GD | DSM-IV | No | Discounting | |
| Kräplin et al., 2015 | 25819174 | Adults | Mixed | Europe | GD | DSM-IV | Yes | SST | |
| Kräplin et al., 2015 | 25819174 | Adults | Mixed | Europe | GD | DSM-IV | Yes | Discounting | |
| Lai et al, 2011 | 22078160 | Adults | Males | Asia | GD | SOGS (5 and over for PG) | No | Stroop | |
| Ledgerwood et al, 2009 | 19615829 | Adults | Mixed | USA | GD | NODS | No | Discounting | |
| Ledgerwood et al., 2012 | 21253846 | Adults | Mixed | Other | GD | NODS | Unclear | Stroop | |
| Leiserson and Pihl, 2007 | 17570042 | Youth | Males | Other | GD | SOGS (5 or over) | Yes | GNG | |
| Lorains et al., 2014 | 24479640 | Adults | Mixed | Other | GD | PGSI (8 or above) | Yes | SST | |
| Mallorquí-Bague et al., 2016 | 27690367 | Adults | Mixed | Europe | GD | SCID-I | No | IGT | |
| Miedl et al., 2015 | 25644499 | Adults | Males | Europe | GD | DSM-IV, KFG, SOGS German | No | Discounting | |
| Mohammadi et al, 2016 | 26239549 | Adults | Males | Europe | GD | SOGS (6 and over for PG) | No | Discounting | |
| Nigro and Cosenza, 2016 | 26879947 | Adolescents | Mixed | Europe | PG | SOGS-RA | Unclear | IGT | |
| Odlaug et al, 2011 | 21426627 | Adults | Mixed | USA | GD | SCI-PG (5 and over for PG) | No | SST | |
| Potenza et al, 2003 | 14594746 | Adults | Males | USA | GD | SOGS, DSM-IV Criteria | No | Stroop | |
| Power et al, 2012 | 22037936 | Adults | Males | Other | GD | SOGS (5 and over for PG) | No | IGT | |
| Regard et al., 2003 | 14765001 | Adults | Mixed | Europe | GD | Unclear | Yes | Stroop | |
| Roca et al., 2008 | 18327015 | Unclear | Unclear | Other | GD | SOGS (5 or over) | No | GNG | |
| Rodriguez-Jimenez et al., 2006 | 16912931 | Adults | Males | Europe | GD | SOGS (Spanish v) (5 and over) | No | SST | |
| Rodriguez-Jimenez et al., 2006 | 16912931 | Adults | Males | Europe | GD | SOGS (Spanish v) (5 and over) | No | GNG | |
| Thomsen et al, 2013 | 23487797 | Adults | Males | Europe | GD | SCID-PG | Yes | SST | |
| Torres et al, 2013 | 23441001 | Adults | Mixed | Europe | GD | IRAB, SCID | No | GNG | |
| Torres et al, 2013 | 23441001 | Adults | Mixed | Europe | GD | IRAB, SCID | No | Discounting | |
| Wiehler et al, 2015 | 26379558 | Adults | Mixed | Europe | GD | SOGS | Unclear | Discounting | |
| Wiehler et al, 2017 | 28612049 | Adults | Males | Europe | GD | SOGS ('Met DSM-IV criteria') | No | Discounting | |
| Wilde et al, 2013 | 26165768 | Adults | Mixed | Europe | GD | SOGS (5 or more for probable pathological gambling) | No | IGT | |
| Wilde et al, 2013 | 26165768 | Adults | Mixed | Europe | GD | SOGS (5 or more for probable pathological gambling) | No | Discounting | |
| Wilde et al, 2013 | 26165768 | Adults | Mixed | Europe | GD | SOGS (5 or more for probable pathological gambling) | No | Stroop | |
| Yan et al, 2016 | 27976705 | Youth | Mixed | Asia | GD | SOGS (5 and over for problem gamblers) | No | Discounting | |
| Zack et al, 2015 | 26152320 | Adults | Males | Other | GD | SOGS (5 and over for PG) | No | SST | |
| Zhou et al, 2016 | 26400106 | Adults | Mixed | Asia | GD | Met DSM-IV criteria | Unclear | GNG | |

## TABLE S2. Studies excluded from the final stage of the data process and reasons.

| Study | PubMed ID | Reason |
| --- | --- | --- |
| van Timmeren et al., 2006 | 27612435 | Domain outside scope (cognitive flexibility). |
| Lawrence et al., 2009 | 19727677 | Insufficient studies for meta-analysis of SST in problem gambling. |
| Verdejo-Garcia et al., 2005 | 26045346 | Domain outside scope (cognitive flexibility). |
| Boog eg al., 2004 | 25165438 | Domain outside scope (cognitive flexibility). |
| de Ruiter et al., 2012 | 21893386 | Insufficient studies for meta-analysis of SST in problem gambling. |
| Lawrence et al., 2009 | 19466924 | Insufficient studies for meta-analysis of CGT or IST in problem gambling; SWM outside scope. |
| Bonini et al., 2008 | 28770486 | Insufficient studies for meta-analysis of BART in GD or problem gambling. |
| Manning et al., 2013 | NA | Insufficient studies for meta-analysis of IST in GD; SOC, IED, outside scope. |
| Grant et al., 2011 | 21715016 | Excluded as overlapping sample with Odlaug et al. |
| Noel et al., 2017 | NA | Excluded as task not in scope (OTSPAN). |
| Cosena et al., 2016 | 27256371 | Insufficient studies to meta-analyse BART or discounting in problem gambling. |

Abbreviations: SST = Stop-Signal tasks; GNG = Go/No-Go tasks; IGT = Iowa Gambling tasks.

## TABLE S3. Quality scores for papers included in the meta-analysis.

| PAPER | GD symptoms assessed using recognized measure (e.g. SOGS; e.g. structured clinical interview) | Report of comorbidities using validated instrument (or excluded based on valid instrument) | Report or substance misuse using appropraite instrument | SUDs actually excluded (based on reasonable steps i.e. clinical interview and/or urine dip) | Report and/or exclusion of ICDs using appropriate instrument | Education and/or IQ | Study reports most appropriate outcome measure(s) | Numerical report (mean, SD, N) within paper (not just graph) | TOTAL |
| --- | --- | --- | --- | --- | --- | --- | --- | --- | --- |
| Aïte et al., 2014 | 1 | 0 | 0 | 0 | 0 | 0 | 1 | 1 | 3 |
| Albein-Urios et al, 2012 | 1 | 1 | 1 | 0 | 0 | 1 | 1 | 1 | 6 |
| Albein-Urios et al, 2014 | 1 | 1 | 1 | 1 | 0 | 1 | 1 | 1 | 7 |
| Álvarez-Moya et al., 2009 | 1 | 0.5 | 0.5 | 1 | 0 | 1 | 1 | 1 | 6 |
| Billieux et al, 2012 | 1 | 1 | 1 | 1 | 0 | 1 | 1 | 1 | 7 |
| Black et al., 2013 | 1 | 1 | 1 | 1 | 1 | 1 | 1 | 1 | 8 |
| Bottesi et al., 2014 | 1 | 0.5 | 0.5 | 0.5 | 0 | 1 | 1 | 1 | 5.5 |
| Brevers et al, 2012 | 1 | 0 | 1 | 1 | 0 | 1 | 1 | 0.5 | 5.5 |
| Brevers et al, 2012a | 1 | 0 | 1 | 1 | 0 | 1 | 1 | 0.5 | 5.5 |
| Brevers et al, 2013 | 1 | 1 | 1 | 1 | 0.5 | 1 | 1 | 1 | 7.5 |
| Brevers et al., 2014 | 1 | 0.5 | 1 | 1 | 0 | 1 | 1 | 1 | 6.5 |
| Cavedini et al, 2002 | 1 | 1 | 1 | 0 | 0 | 1 | 1 | 1 | 6 |
| Choi et al, 2014 | 1 | 0.5 | 0.5 | 0 | 0 | 1 | 1 | 1 | 5 |
| Ciccarelli et al, 2016 | 0 | 0 | 0 | 1 | 0 | 0 | 1 | 1 | 3 |
| Ciccarelli et al, 2017 | 1 | 0 | 1 | 1 | 0 | 1 | 1 | 0.5 | 5.5 |
| Contreras-Rodriguez et al, 2015 | 1 | 1 | 1 | 1 | 0.5 | 1 | 1 | 1 | 7.5 |
| Forbush et al., 2008 | 1 | 1 | 0.5 | 0 | 0 | 1 | 1 | 1 | 5.5 |
| Fuentes et al., 2006 | 1 | 1 | 1 | 0 | 0 | 1 | 1 | 1 | 6 |
| Goudriaan et al., 2006 | 1 | 1 | 1 | 1 | 0.5 | 1 | 1 | 1 | 7.5 |
| Hur et al, 2012 | 1 | 0 | 1 | 1 | 0 | 1 | 1 | 1 | 6 |
| Kalechstein et al., 2007 | 0 | 1 | 1 | 1 | 1 | 1 | 0.5 | 1 | 6.5 |
| Kapsomenakis et al, 2018 | 1 | 0 | 0 | 0 | 0 | 1 | 1 | 1 | 4 |
| Kertzman et al., 2006 | 1 | 0 | 0 | 1 | 0 | 1 | 1 | 1 | 5 |
| Kertzman et al., 2007 | 1 | 0.5 | 1 | 1 | 1 | 1 | 1 | 1 | 7.5 |
| Kertzman et al., 2011 | 1 | 0.5 | 0.5 | 1 | 0 | 1 | 0.5 | 0.5 | 5 |
| Kräplin et al., 2014 | 1 | 1 | 1 | 1 | 0.5 | 1 | 1 | 1 | 7.5 |
| Kräplin et al., 2015 | 1 | 1 | 1 | 1 | 0.5 | 1 | 1 | 1 | 7.5 |
| Lai et al, 2011 | 1 | 1 | 1 | 0 | 0 | 1 | 0.5 | 1 | 5.5 |
| Ledgerwood et al, 2009 | 1 | 0 | 0 | 1 | 0 | 1 | 1 | 1 | 5 |
| Ledgerwood et al., 2012 | 1 | 1 | 1 | 1 | 0 | 1 | 1 | 0.5 | 6.5 |
| Leiserson and Pihl, 2007 | 1 | 1 | 1 | 1 | 0.5 | 1 | 1 | 1 | 7.5 |
| Lorains et al., 2014 | 1 | 1 | 1 | 0 | 0 | 1 | 1 | 1 | 6 |
| Mallorquí-Bague et al., 2016 | 1 | 1 | 1 | 1 | 0 | 1 | 1 | 1 | 7 |
| Miedl et al., 2015 | 1 | 0 | 0 | 0 | 0.5 | 1 | 1 | 1 | 4.5 |
| Mohammadi et al, 2016 | 1 | 1 | 1 | 1 | 0 | 1 | 1 | 1 | 7 |
| Nigro and Cosenza, 2016 | 1 | 0 | 0 | 0 | 0 | 0 | 1 | 1 | 3 |
| Odlaug et al, 2011 | 1 | 1 | 1 | 1 | 1 | 1 | 1 | 1 | 8 |
| Potenza et al, 2003 | 1 | 1 | 1 | 1 | 0 | 1 | 1 | 1 | 7 |
| Power et al, 2012 | 1 | 1 | 1 | 1 | 0 | 0 | 1 | 1 | 6 |
| Regard et al., 2003 | 0 | 0 | 0 | 1 | 0 | 0 | 0.5 | 1 | 2.5 |
| Roca et al., 2008 | 1 | 0 | 1 | 1 | 0 | 1 | 1 | 0 | 5 |
| Rodriguez-Jimenez et al., 2006 | 1 | 0.5 | 0.5 | 1 | 0 | 1 | 1 | 1 | 6 |
| Thomsen et al, 2013 | 1 | 1 | 1 | 0 | 0 | 0 | 1 | 1 | 5 |
| Torres et al, 2013 | 1 | 1 | 1 | 1 | 0 | 1 | 1 | 1 | 7 |
| Wiehler et al, 2015 | 1 | 0 | 0.5 | 0.5 | 0 | 1 | 1 | 0 | 4 |
| Wiehler et al, 2017 | 1 | 0 | 1 | 0 | 0 | 1 | 1 | 0 | 4 |
| Wilde et al, 2013 | 1 | 1 | 1 | 1 | 0 | 1 | 0.5 | 1 | 6.5 |
| Yan et al, 2016 | 1 | 1 | 1 | 1 | 0 | 0 | 1 | 1 | 6 |
| Zack et al, 2015 | 1 | 1 | 1 | 1 | 0 | 0 | 1 | 1 | 6 |
| Zhou et al, 2016 | 0 | 0 | 0 | 0 | 0 | 1 | 1 | 1 | 3 |

## FIGURE S1 - Funnel Plots


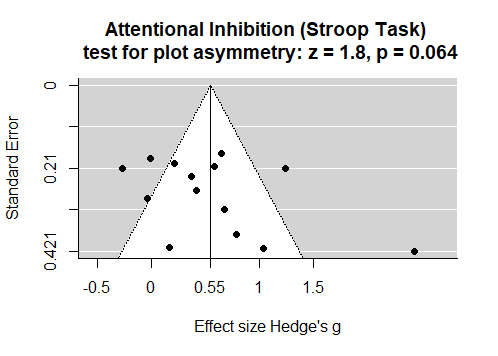

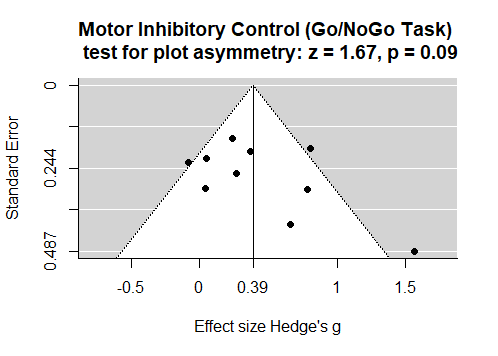

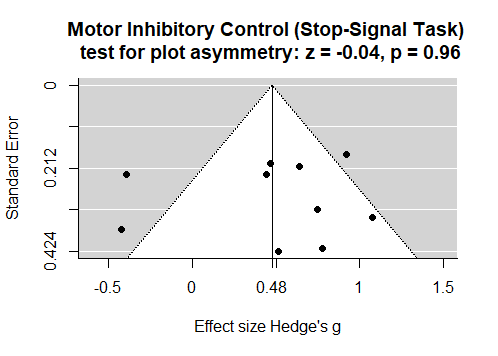

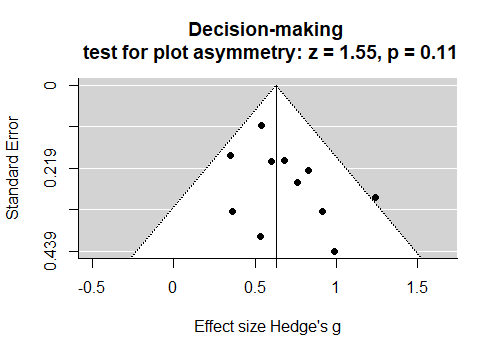

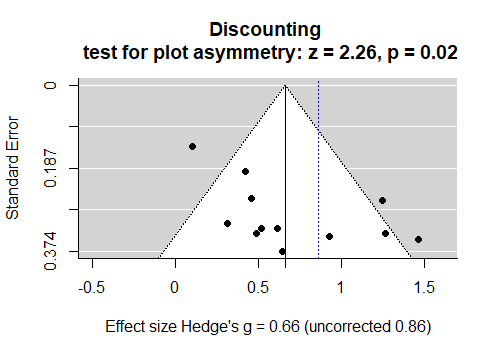

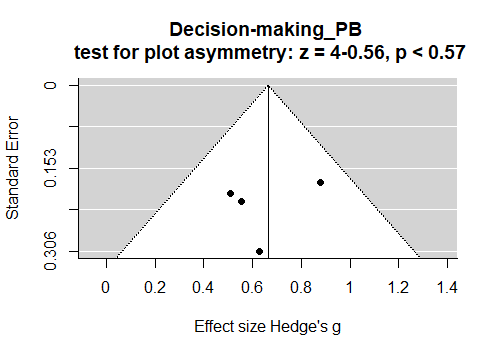


STROOP = Stroop task; GNG = Go/No-Go task; SST = Stop-signal task; DM = Decision-making tasks; DISC = Discounting taks; DM_PB = Decision-making in problem gamblers

## TABLE S4 - Heterogeneity and model estimate measures for different cognitive domains

| Domain | N studies | tau^2 (se) | I^2 | H^2 | Q-test  (p value) † | Model Estimate (se) |
| --- | --- | --- | --- | --- | --- | --- |
| Attentional Inhibition (Stroop) | 14 | 0.29(0.14) | 83.1% | 5.94 | **<0.0001** | 0.55 |
| Motor inhibitory control (GNG) | 10 | 0.08(0.07) | 59.1% | 2.45 | **0.007** | 0.39 |
| Motor inhibitory control (SST) | 10 | 0.17(0.11) | 72.0% | 3.57 | **0.0002** | 0.48 |
| Decision-Making | 11 | 0.003(0.02) | 4.71% | 1.05 | 0.39 | 0.63 |
| Discounting | 14 | 0.46(0.21) | 85.7% | 7.01 | **<0.001** | 0.66 |
| Decision-making in problem Gambling | 4 | 0.002(0.03) | 3.06% | 1.03 | 0.50 | 0.66 |

tau^2: estimated amount of total heterogeneity; I^2: (total heterogeneity / total variability); H^2: (total variability / sampling variability); Q-test: Test for Heterogeneity; meta-analysis was done using random-effects model using REML. REML: Restricted maximum-likelihood estimator

## TABLE S5 - Moderation analysis table

|  | Age | Gender | Geographical | Co-morbidities | Quality |
| --- | --- | --- | --- | --- | --- |
| STROOP | Redundant | 0.81 | 0.64 | 0.92 | 0.24 |
| GNG | 0.064 | 0.30 | **0.010*** | 0.73 | 0.27 |
| SST | Redundant | **0.003**** | 0.28 | 0.28 | **0.029*** |
| DISC | **0.03*** | 0.97 | **0.007**** | 0.42 | 0.85 |
| DM | Redundant | 0.96 | 0.29 | 0.31 | 0.12 |
| DM_PB | 0.31 | 0.34 | 0.48 | Redundant | 0.74 |

STROOP = Stroop task; GNG = Go/No-Go task; SST = Stop-signal task; DM = Decision-making tasks; DISC = Discounting taks; DM_PB = Decision-making in problem gamblers; In GNG, effect sizes varied by geographical area Asian < Europe; In SST mixed gender report higher estimates (0.71 vs -0.02 intercept); higher quality studies reporting bigger effect sizes. In DISC adult studies reported higher estimates than the youth study (Yan et al); effect sizes varied by geographical area Asian < Europe < USA. Redundant = model did not have enough variable categories to perform moderation analysis. Studies excluded as outliers (Cook’s d influence > 2 s.d and Quality score < 15%) were Regard et al 2003 (Stroop task) and Weihler et al, 2017 (Discounting). Significance p < 0.05 = ‘*’; p<0.01 = ‘**’; p<0.001= ‘***’.

## TABLE S6 - Moderation without exclusion of outliers

|  | Age | Gender | Geographical | Co-morbidities | Quality |
| --- | --- | --- | --- | --- | --- |
| STROOP | Redundant | 0.57 | 0.55 | 0.051 | **0.0019 **** |
| GNG | 0.064 | 0.30 | **0.010*** | 0.73 | 0.27 |
| SST | Redundant | **0.003**** | 0.28 | 0.27 | **0.029*** |
| DISC | 0.24 | 0.31 | 0.48 | 0.45 | 0.34 |
| DM | Redundant | 0.96 | 0.29 | 0.31 | 0.12 |
| DM_PB | 0.31 | 0.34 | 0.48 | Redundant | 0.74 |

STROOP = Stroop task; GNG = Go/No-Go task; SST = Stop-signal task; DM = Decision-making tasks; DISC = Discounting taks; DM_PB = Decision-making in problem gamblers; In GNG, effect sizes varied by geographical area Asian < Europe; In SST mixed gender report higher estimates (0.71 vs -0.02 intercept); higher quality studies reporting bigger effect sizes. In DISC adult studies reported higher estimates than the youth study (Yan et al); effect sizes varied by geographical area Asian < Europe < USA. Redundant = model did not have enough variable categories to perform moderation analysis.

## FIGURE S2 – Forest, funnel and influence plots without the exclusion of outliers

### Figure S2a – Stroop (attentional inhibition) domain forest, influence and funnel plot, without exclusion of Regard et al. 2003


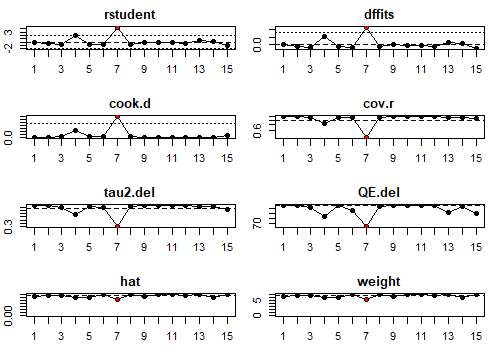

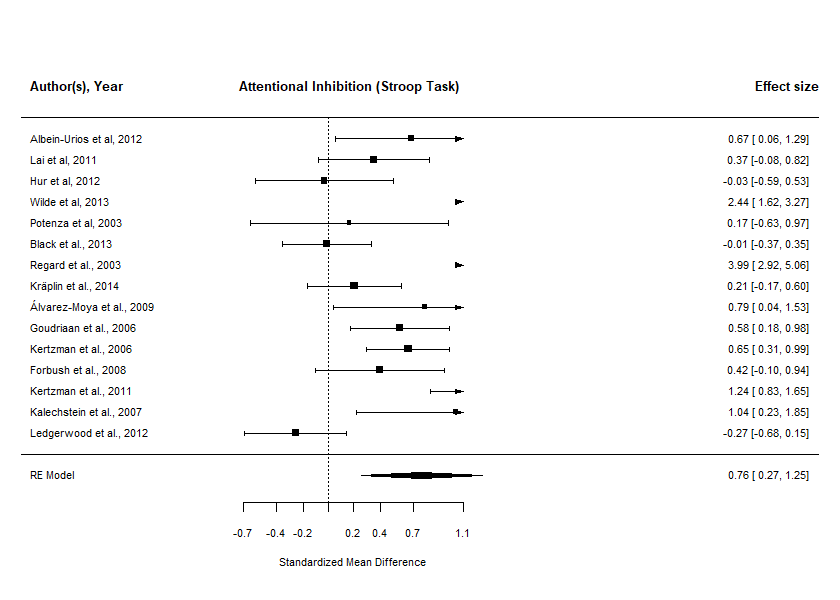

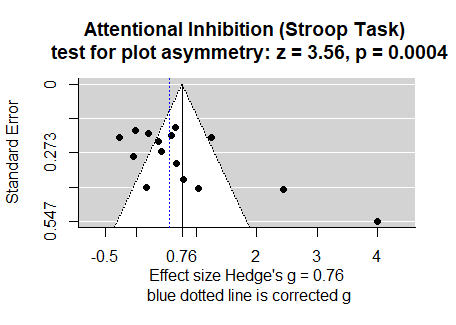


###
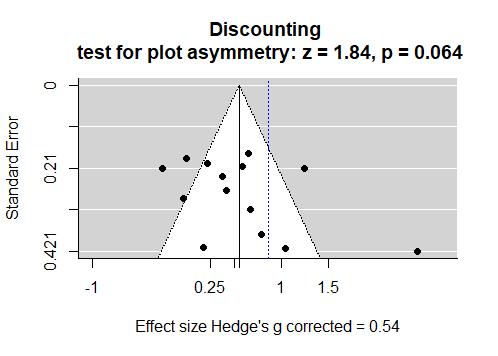

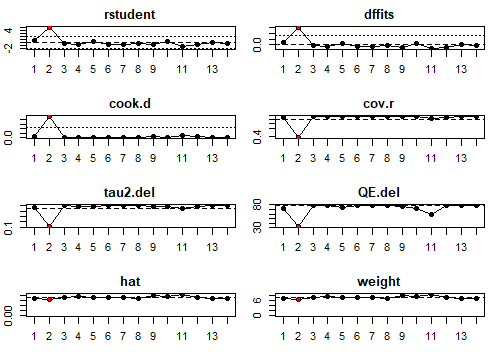
Figure S2b – Discounting domain forest, influence and funnel plot, without exclusion of Wiehler et al. 2017
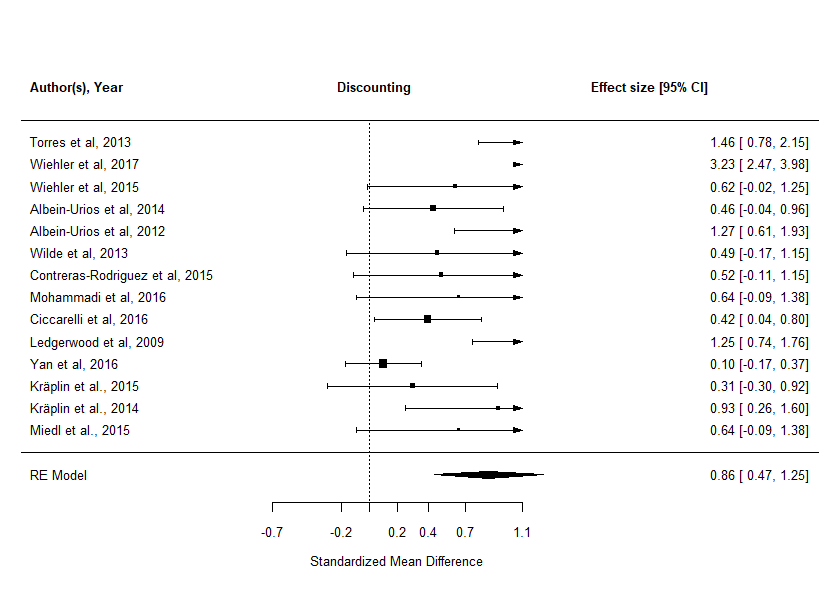


### Figure S2c – Decision-making in problem gamblers forest, influence and funnel plot
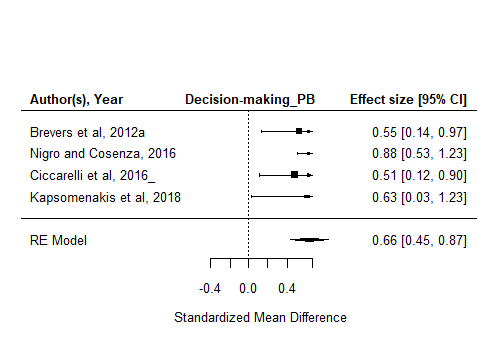

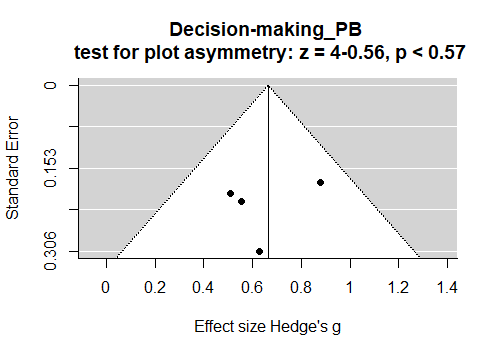

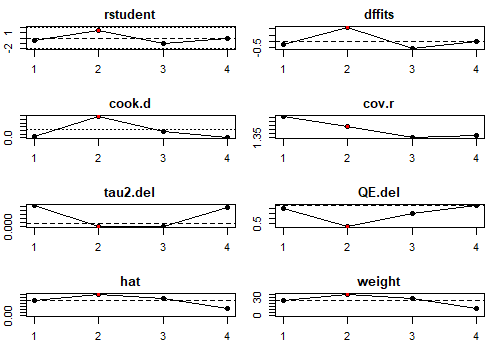


## R code

library(dplyr); library(data.table); library("robumeta"); library("metafor")

# We are following the analyses described in Quintana 2015, the data is included with the metafor package. Use the following the command to load the data. You are creating a new object called "df". Data manipulation with dplyr and data.table

df <- read.csv("~/Psychiatry Research/Gambling/df.csv", head = TRUE, stringsAsFactors = FALSE); df <- data.table(df)df <- df %>% select(Task, ID, paper, piu.mean:control.N)#, Age:Co.morbidities) # This selects relevant variables

df_Stroop <- df %>% filter(Task == "Stroop") %>%

dplyr::select(ID, paper, piu.mean:control.N) %>%

filter(complete.cases(.))# %>%

#filter(ID != "7") (to exclude outlier study); this is run after the influence analysis

# The first step is to transform r to Z and calculate the corresponding sample variances.(Quintana) "SMD" is measure outcome for the standardized mean difference from the escalc function #slab is an optional vector with labels for the studies

df_Stroop <- escalc(measure="SMD", m1i=piu.mean, m2i=control.mean, sd1i=piu.SD, sd2i=control.SD, n1i=piu.N, n2i=control.N,

data=df_Stroop, slab=paper)

# Now you're ready to perform the meta-analysis using a random-effects model. The following commands will print out the data and also calculates and print the confidence interval for the amount of heterogeneity (I^2).

res_Stroop <- rma(yi, vi, data=df_Stroop)

res_Stroop

# While the Q-statistic and I^2 can provide evidence for heterogeneity, they do not provide information on which studies may be influencing to overall heterogeneity. If there is evidence of overall heterogeneity, construction of a Bajaut plot can illustrate studies that are contribute to overall heterogeneity and the overall result. Study IDs are used to identify studies # A set of diagnostics are also available to identify potential outliers and influential cases.

inf <- influence(res_Stroop) #in this particular case we see Regard as highly influential case

print(inf); plot(inf) # The plot visualizes the printed dataset. As there are no studies are marked with an asterisk in the printed dataset, none of the studies fulfilled the criteria as an influential study.

# Now we visualize the meta-analysis with a forest plot.

par(font=1)

forest(res_Stroop, xlim=c(-2.5,2.5),

at=transf.rtoz(c(-0.6,-.4,-.2,0,.2,.4,.6, .8)), digits=c(2,1), cex=.7)

par(font=2, cex=1)

text(-2.5, 16.5, "Author(s), Year", pos=4, cex=.8)

text(-0.8, 16.5, "Attentional Inhibition (Stroop Task)", pos=4, cex=.8)

text( 2.5, 16.5, "Effect size [95% CI]", pos=2, cex=.8)

#We've created a plot with all studies. Importantly, the correlations and 95% CIs are reported for each study as well as the summary effect size (the polygon at the bottom). The edges of the polygon represent the 95% confidence limit. Note the different sizes of each square - the studies with larger squares contributed more to the summary effect size.

### funnel plot

par(font=1, cex=1)

funnel(res_Stroop, xlab = "Effect size Hedge's g = 0.76\n blue dotted line is corrected g", at= c(-0.5, 0, as.numeric(res_Stroop$beta), 1, 2, 3, 4),

main= "Attentional Inhibition (Stroop Task) \n test for plot asymmetry: z = xxx, p = xxx")

abline(v=as.numeric(0.55), col = "blue", lty = "dotted")

res.tf <- trimfill(res_Stroop)

funnel(res.tf, xlab = "Effect size Hedge's g",

main= "Stroop \n test for plot asymmetry: z = xxx, p = xxx",

at= c(-3, -2, -1, 0, as.numeric(res.tf$beta), 0.5, 1))

abline(v=as.numeric(res_Stroop$beta), col = "blue", lty = "dotted")

res.tf

#Tests for bias

regtest(res_Stroop); ranktest(res_Stroop)

#Neither Egger's regression test or the Rank correlation test was statistically significant so there's no evidence of publication bias according to these tests.

#And we repeat for all other domains

#Now we do moderation analysis

df <- read.csv("~/Psychiatry Research/Gambling/df.csv", head = TRUE, stringsAsFactors = FALSE); df <- data.table(df)

df <- df %>% select(Task, ID, paper, piu.mean:control.N, Age:Co.morbidities) ; df$Age <- as.factor(df$Age); df$Co.morbidities <- as.factor(df$Co.morbidities)

dfm_Stroop <- df %>% filter(Task == "Stroop") ;dfm_Stroop <- escalc(measure="SMD", m1i=piu.mean, m2i=control.mean, sd1i=piu.SD, sd2i=control.SD, n1i=piu.N, n2i=control.N, data=dfm_Stroop, slab=paper)

# Moderating effect of age (all use a meta-regression model)

res.modage <- rma(yi, vi, mods = ~ Age, data=dfm_Stroop) ; res.modage

res.modgender <- rma(yi, vi, mods = ~ Gender, data=dfm_Stroop) ; res.modgender

res.modgeo <- rma(yi, vi, mods = ~ Geographical, data=dfm_Stroop) ;res.modgeo

res.modmorb <- rma(yi, vi, mods = ~ Co.morbidities, data=dfm_Stroop) ; res.modmorb

res.modmorb <- rma(yi, vi, mods = ~ Quality.score, data=dfm_Stroop) ; res.modmorb
